# Open data 5 years on: a case series of 12 freedom of information requests for regulatory data to the European Medicines Agency

**DOI:** 10.1186/s13063-016-1194-7

**Published:** 2016-02-11

**Authors:** Peter Doshi, Tom Jefferson

**Affiliations:** Department of Pharmaceutical Health Services Research, University of Maryland School of Pharmacy, 220 Arch St, Floor 12, Rm. 01-228, Baltimore, MD 21201 USA; Cochrane Acute Respiratory Infections Group, 00187 Rome, Italy

**Keywords:** Freedom of information act, Freedom of information, Access to documents, CSR, European medicines agency, Clinical study report, EMA, Regulatory science, Systematic reviews, Cochrane collaboration, Reporting bias, Evidence synthesis, Publication bias

## Abstract

**Background:**

Clinical trial (and other) data from the European Medicines Agency (EMA) offers the best available opportunity to address the extensive reporting bias in pharmaceutical trial literature. Data are requested via freedom of information requests, but 5 years on, little is known about how the system is working.

**Methods:**

Case series of 12 requests for regulatory data (clinical study reports and other regulatory data) relating to 29 different compounds. We logged start and end dates for correspondence with and data releases from the EMA, the need for additional correspondence and appeal of initial negative decisions, and inspected data releases for redaction. We measured: time from initial request to first substantive response from the EMA, to final decision from the EMA (in case of appeal), to initial receipt of documents, and to completion of request; number of data transmission batches generated; number of pages received for each request; average number of pages per batch over time (for releases in multiple batches); judgment as to whether the request was satisfied.

**Results:**

We found great variability in time to receive an initial decision from the EMA (1 to 13 weeks). Additional correspondence with the EMA was necessary in 10 of 12 requests. Four of 12 were initially refused but 3 of 4 were allowed on appeal after 3 to 33 additional weeks. One request was denied despite appeal. Time to final decision was 1 to 43 weeks. We received data for 11 of 12 requests in 98 batches. While two requests remain outstanding as at June 2015 the remaining nine requests took a median 43 weeks to completion (range: 17 to 186 weeks). Despite redaction in 10 of 11 releases (mainly of researcher and participant identifying information), 8 requested were wholly satisfied.

**Conclusions:**

The EMA is the only regulator in the world that is routinely releasing original clinical trial data, but release can take considerable time to occur and often only after a lengthy correspondence. Given its importance for research and significance for transparency we suggest ways in which the process could be made more efficient.

**Electronic supplementary material:**

The online version of this article (doi:10.1186/s13063-016-1194-7) contains supplementary material, which is available to authorized users.

## Background

For those engaged in research synthesis, regulatory data not traditionally publicly available are a crucial weapon to avoid or minimize the impact of reporting bias. These data, typified by clinical study reports ranging from hundreds to thousands of pages, provide a wealth of auditable details on clinical trials well beyond the most extensive journal publications [1].

While calls to address the many problems of reporting biases go back decades [2], the past 5 years have witnessed notable progress in the range of campaigns [3, 4], policies [5, 6], statements and structures [7–11] established to expand public access to clinical trial data.

Among all the data transparency initiatives, one process remains unique: that of the European Medicines Agency (EMA). Since November 2010, the EMA as data holder has been providing requestors with clinical trial data relating to compounds on which a decision has been made under the centralized registration procedure [5].

The EMA has two distinct policies affecting public access to clinical trial data in its holdings, one on request [12, 13] and the second for data in marketing authorization applications (MAAs) regarding MAAs submitted on or after 1 January 2015 (with web data release to start in 2016) [14].

Since 2010, the EMA has released over two million pages of regulatory data on request [15, 16], mainly to industry [16]. The number of requests has grown over time, from 20 requests per month during the first 2 years to nearly double that in the 6-month period after, but pages released in this time period decreased from around 70,000 pages per month to 44,000 [16].

In September 2013, the EMA expanded its ability to deal with the growing number of requests, dedicating a full single 12-person team to a new “Access to Documents Service” (ATD). When the service began in 2010, five EMA staff who had other job responsibilities unrelated to disclosure handled all requests. Following the reorganization, the new ATD team had 12 full-time employees (personal communication, Anne-Sophie Henry-Eude, 17 November 2014). In 2014, the EMA also released a guide to help requestors [17].

However, as repeated requestors and recipients of data from the EMA, our experience has been one of increasing delay and complexity.

We report on the details and outcome of 12 requests for regulatory data made by us to the EMA between 2011 and June 2015.

## Methods

### Types of requests

We examined all requests we submitted to the EMA between November 2010 (when the new EMA policy went into force) and 30 May 2015, regardless of their fate (acceptance, partial rejection, or complete rejection). We included all requests for trial data (the text of the trial itself with its protocol and amendments, analysis plans and tables of results – the clinical study report – and other documents used by regulators related to trials such as parts of submissions for the MAA based on trials). We included animal study data. We treated each request as a separate case, thereby constructing our own case series of requests.

Following the submission and receipt of a request, the Agency may additionally seek clarification, such as which specific data are being requested, and in what priority. If the EMA decides to grant the request, it will consult with the marketing authorization holder of the relevant product prior to release of data. If the EMA rejects a request, it allows the requestor 15 working days to appeal this decision. The EMA may grant certain portions of a request, but reject others (again, allowing appeal).

We extracted information across two domains: correspondence and data releases.

### Extraction of correspondence

We logged all electronic correspondence and linked data releases to us from the EMA. We recorded the type of documents requested, the initial date of each request and the date of initial EMA approval or rejection of each request.

We extracted and tabulated data on a simple spreadsheet on the object and date of the initial request and answer, in what professional capacity we made the request (academic researcher or journalist), the presence, count and timelines of appeals and their resolution, and whether additional correspondence took place including clarifications, request modifications and prioritization of releases from a list of EMA holdings.

### Extraction of data releases

We recorded the number of pages released and total number of batches (release of data on a given date) in which documents were released. Larger requests are typically split into multiple batches with no single batch fulfilling the scope of the overarching request.

We extracted the start and finish date of each data release. For requests ongoing as of 1 June 2015, we used the most recent data release date prior to this date. We also recorded presence and type of redactions and omission of entire sections of clinical study reports.

### Analysis

We calculated the length of time from initial request to: initial decision by the EMA, final decision (in case of appeals), initial receipt of documents, and completion of request (or most recent data release, in case of ongoing requests).

We calculated the number of data transmission batches our requests generated, the total number of pages for each request and the average number of pages per batch over time, for requests that were split into multiple batches.

Finally, we made a subjective judgment as to whether our request was satisfied (i.e., what we received matched what we requested), recorded as either “Yes,” “Mostly,” or “No.”

We did not seek ethical approval of our research as it is not research on human subjects.

## Results

We made 15 freedom of information requests to the EMA between January 2011 (the date of our earliest request) and 1 June 2015 (our cutoff date). Three requests were excluded from our analysis (two for correspondence unrelated to trial data and one because of submission only 4 days prior to our cutoff date). One request was for documents relating to an EMA review of safety data from a single company on a range of its products [18]. The remaining 11 requests related to 29 compounds: 18 antibiotics, 2 antivirals, 1 monoclonal antibody, 4 vaccines, 1 volume expander, 1 antipsychotic, 1 statin, and 1 antidiabetic (Table 1). Nine of the 12 requests were made in our capacity as academic researchers; 3 were made in our capacity as journalists.

**Table 1:** Table of 12 included requests in chronological order

Request no.	Date of request	Compounds	Documents requested
1	25 Jan 2011	1 antiviral: oseltamivir	31 CSRs
2	29 Oct 2011	2 vaccines: Pandemrix and Focetria	4 CSRs, regulatory comments, decision records, other material such as slides, correspondence, records of meetings, follow-up material
3	22 Dec 2011	1 antipsychotic: aripiprazole	3 CSRs
		1 statin: atorvastatin	
		1 antidiabetic: pioglitazone	
4	11 Sep 2012	1 antiviral: oseltamivir	38 animal study reports
5	23 Oct 2012	1 antiviral: oseltamivir	3 periodic safety update reports
6	2 Dec 2012	1 antiviral: oseltamivir	Specific analysis from ICH CTD module 5
7	25 Feb 2013	17 antibiotics^a^	Multiple sections of CSR from 97 CSRs
8	4 Apr 2014	1 anti-inflammatory: adalimumab	3 CSRs
9	16 Apr 2014	1 antibiotic: bedaquiline	3 CSRs, CHMP minutes and other CHMP documents
10	16 Apr 2014	48 medicines [21]	Infringement procedure documents
11	28 May 2014	2 vaccines: Gardasil and Cervarix	43 CSRs
		1 antiviral: sofosbuvir	
12	8 May 2015	1 volume expander: hydroxyethyl starch	Postmarketing data submitted by the manufacturer

^a^Altargo, Cayston, Colobreathe, Cubicin, Dificlif, Doribax, Invanz, Ketek, Levviax, Tobi Podhaler, Trovan, Trovan IV, Truvel, Truvel IV, Tygacil, Vibativ, Zinforo

*CHMP* Committee for Medicinal Products for Human Use, *CSR* Clinical Study Report, *CTD* Common Technical Document, *ICH* International Conference on Harmonization of Technical Requirements for Registration of Pharmaceuticals for Human Use

Table 2 shows the great time variability to receive an initial decision from the EMA (1 to 13 weeks). Ten of 12 requests entailed additional correspondence with the EMA beyond the initial request, for example to clarify the scope of the EMA’s holdings for a particular therapeutic.

**Table 2:** Timeline of correspondence milestones

Request no.	Documents received^b^	Time to initial decision, weeks	Time to final decision (if appeal lodged), weeks	Time to initial receipt of data, weeks	Time to final receipt of data, weeks
1	16 CSRs	2	N/A	9	17
2	4 CSRs, regulatory comments, decision records, other material such as slides, correspondence, records of meetings, follow-up material	4	N/A	22	186
3	8 CSRs	9	N/A	9	33
4	38 animal study reports	4	N/A	3	17
5	3 periodic safety update reports	1	N/A	5	36
6	Specific analysis from ICH CTD module 5	10	43	43	43
7	Multiple sections of CSR from 97 CSRs	13	22	58	112
8	3 CSRs	7	N/A	7	49
9	3 CSRs, CHMP minutes and other CHMP documents	11	N/A	16	49
10	None	3	7	Appeal denied	
11^a^	43 CSRs for 2 vaccines and 1 antiviral	4	7	15	47
12^a^	Postmarketing data submitted by the manufacturer	3	N/A	3	3

^a^release still ongoing at cutoff date; ^b^may include items expected but not received as of cutoff date

*CHMP* Committee for Medicinal Products for Human Use, *CSR* Clinical Study Report, *CTD* Common Technical Document, *ICH* International Conference on Harmonization of Technical Requirements for Registration of Pharmaceuticals for Human Use, *N*/*A* not applicable

After clarification, 4 of the 12 requests were initially rejected. Reasons included then-active legal proceedings and confidentiality regarding an ongoing regulatory procedure. Examples of request, rejection, and appeal letters can be seen at http://www.bmj.com/tamiflu/ema/. We appealed in all cases, and received a positive decision for three of four appeals in 3 to 33 additional weeks. The time to receive a final decision, therefore, took between 1 and 43 weeks.

In total, the EMA sent us data in response to 11 of 12 requests. The EMA began sending data for half of requests within 9 weeks. However, for the other half, it took between 15 and 58 weeks before any data were received. Two requests were outstanding at our cutoff date. Of the remaining nine requests, it took a median 43 weeks to between initial request to final receipt of data (range: 17 to 186 weeks) (Table 2).

**Fig. 1:**
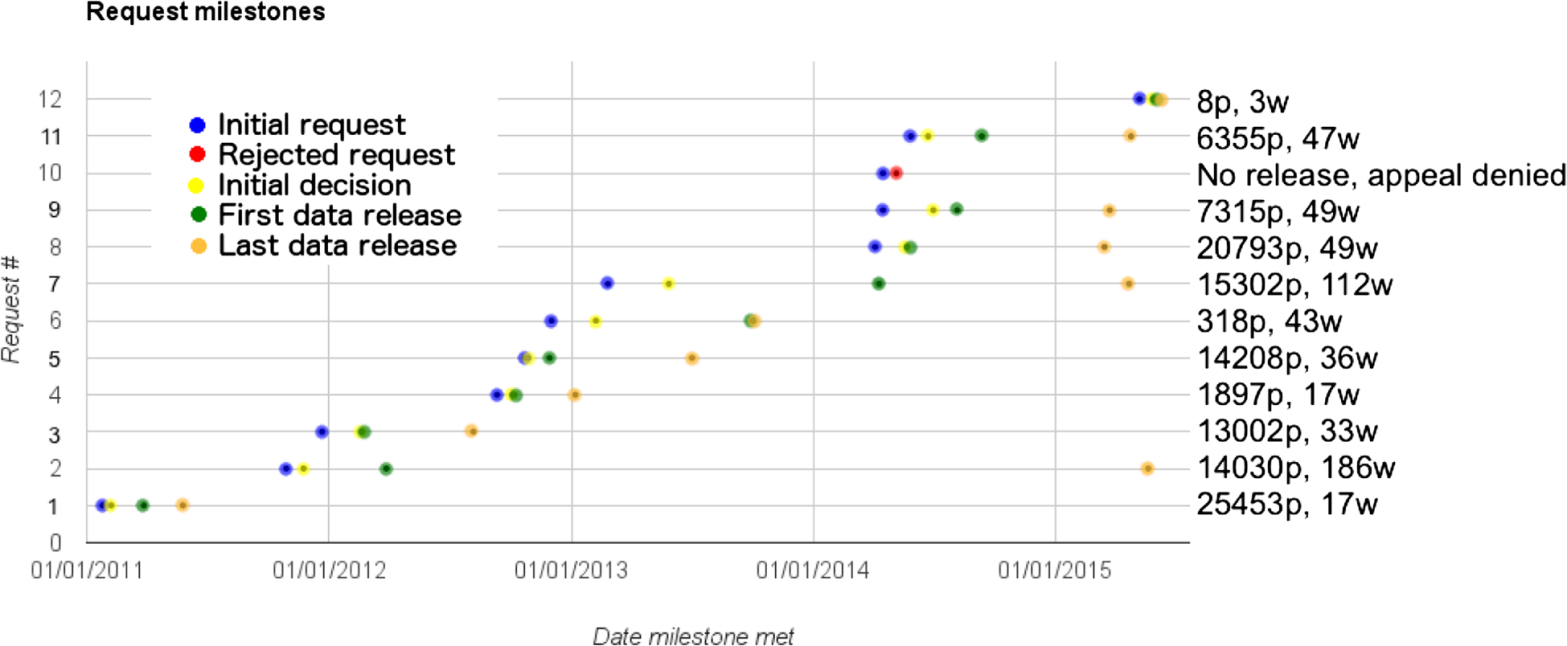
Timeline of 12 requests along four milestone dates (initial request, initial decision, initial release of data, and final release of data). Data points indicate, from left to right: initial request, initial decision, initial release of data, and final release of data

There was great variability in the length of released documents. Clinical study reports ranged from 7315 to 25,453 pages. Releases delivered in batches were divided into no more than 5 batches before the year 2013, but afterwards were divided into between 10 and 22 batches. We observed no relationship between the overall number of pages released and number of batches but requests since 2013 were generally delivered in a greater number of smaller batches. There was no relationship between the number of batches and number of clinical study reports. Our first request was released in 4 batches and contained a total of 16 clinical study reports (average 6363 pages per batch), whereas our eighth request for 3 clinical study reports was delivered across 12 batches (average 1733 pages per batch) (Table 3; Fig. 1).

**Table 3:** Released documents

Request no.	Request	Pages	Batches	Pages per batch	Redacted?	Items missing?	Request satisfied?
1	1 antiviral: oseltamivir	25,453	4	6363	No	No	Yes
2	2 vaccines: Pandemrix and Focetria	14,030	4	3508	Yes	Yes	Mostly
3	1 antipsychotic: aripiprazole	13,002	4	3251	Yes	Not sure	Yes
	1 statin: atorvastatin						
	1 antidiabetic: pioglitazone						
4	1 antiviral; oseltamivir	1897	3	632	Yes	No	Yes
5	1 antiviral: oseltamivir	14,208	5	2842	Yes	Not sure	Yes
6	1 antiviral: oseltamivir	318	1	318	Yes	No	No
7	13 antibiotics^a^	15,302	21	729	Yes	No	Yes
8	1 anti-inflammatory: adalimumab	20,793	12	1733	Yes	Yes	Mostly
9	1 antibiotic: bedaquiline	7315	10	732	Yes	Yes	Yes
10	48 medicines	No release					No
11	2 vaccines; Gardasil and Cervarix	6355	22	289	Yes	Yes	Yes
	1 antiviral: sofosbuvir						
12	1 volume expander: hydroxyethyl starch	8	1	8	Yes	No	Yes

^a^Altargo, Cayston, Colobreathe, Cubicin, Dificlif, Doribax, Invanz, Ketek, Tobi Podhaler, Trovan, Tygacil, Vibativ, Zinforo

Only our first request was released without any redaction. All others had variable amounts and types of redactions. Names and other potentially identifying details of researchers and participants (e.g., participant ID number) was the most common—but inconsistently applied—type of redaction. Other details redacted in some cases included procedures or methods (e.g., histology testing details), formulation lot numbers, and the ID numbers of other studies. Some redactions were so extensive that we were unable to guess what information was redacted. Despite the redactions, we judged that in 8 of 12 cases the releases matched our request, 2 cases of partial match, and 2 cases of no match.

The dataset underlying this analysis is available, in Microsoft Excel format, as an online supplement (see Additional File 1).

## Discussion

With little exception, the US Food and Drug Administration treats clinical study reports and other parts of the dossier submitted by sponsors as commercial confidential information and, therefore, not releasable under the US Freedom of Information Act. In contrast, the EMA interprets all documents, including clinical study reports, to be subject to its “reactive” freedom of information policy and is the only regulator in the world routinely releasing such data. However, the agency is dealing with a huge and growing number of requests [15, 16].

We made our first request to the EMA in January 2011, 2 months after the promulgation of its then new policy. We requested clinical study reports for inclusion in our Cochrane review of neuraminidase inhibitors [19]. Four months later, we reached this goal, receiving 16 unredacted clinical study reports for oseltamivir. These documents offered a wealth of detail greatly surpassing any journal-based trial publication. We became quick advocates of using regulatory data for research synthesis because of its powerful potential to address reporting bias in journal publications [20].

However, as time passed we also witnessed considerable heterogeneity in times and complexity of the request process while batches tended to become smaller and fulfillment of requests took longer. Whereas in 2011, we received 16 clinical study reports (25,453 pages) across 4 batches in 4 months, a recent request for clinical study reports for another product has so far been delivered across 22 batches in almost a year, with an average of 289 pages per batch. The fewer pages per batch imposes an administrative burden on requestors as clinical study reports may be split across multiple files and multiple batches but must be manually combined, to create a complete report, necessary to carry out an assessment. Second, the tight appeal time (15 working days) imposes a fair and timely evaluation of whether redactions are reasonable for each batch of documents. We found this task hard to keep up with. We suggest keeping a careful and up-to-date request log to avoid losing data or duplicating the request, both of which happened to us.

Long data access request and receipt timelines may provide an obstacle for researchers accustomed to readily accessible journal publications and encourage neglect of what is as near a “gold standard” of evidence completeness as is currently possible. Some researchers may choose to forgo a chance to address reporting bias in their work and in doing so perpetuate the known problems in contemporary scientific publications.

A second concern is that the length of the EMA’s process may lead some to argue against the EMA’s approach itself and in favor of other data access initiatives such as ClinicalStudyDataRequest.com, a joint initiative of 12 pharmaceutical companies. While we support all efforts in this area, the EMA’s approach remains unique, and is based on the view that it is the regulator’s duty to make available data underlying decision-making for all drugs in its purview. We concur, and believe this approach to be self-evident for any scientific body operating within a political democracy as the EMA does not screen requests by requiring a reason for accessing the data (but a rationale for access when appealing a negative decision is prudent).

We remain firm advocates of broader use of clinical study reports but users should also be aware of EMA-unrelated complexities in the most basic first step: identifying a trial. In some cases, a trial can have up to four identifiers: two different alphanumeric identifiers given by the manufacturer, a registry number and sometimes an acronym. Because the EMA, the manufacturer and registry operators do not necessarily provide tables of holdings that cross-reference these IDs, this can be a further source of confusion and resource wastage.

Over time, CSRs available under the EMA’s “reactive” policy may become less important as they relate to progressively older pharmaceuticals and the EMA’s “prospective” policy of publishing clinical trial data applies to a greater number of drugs in current use. However, the EMA’s reactive policy will remain of great interest for the foreseeable future, especially should the prospective policy falter.

Simple measures may at least partially address some of the problems we identified. First, a permanent forum between the EMA and requestors may help each side maximize the efficiency of the data request/release process through mutual education and exchange of views.

Second, a publicly available list of holdings by compound name would focus requests and avoid unnecessary correspondence in identifying desired materials. On several occasions the EMA offered us a list of its holdings relating to our requests. These proved invaluable in prioritizing and focusing our requests. Although these lists were custom-created for our requests, publicly available lists across EMA holdings would help clarify requests up front and moderate expectations. One tangible way to support the EMA’s efforts would be to call for volunteer researchers to help build a list of EMA holdings, perhaps in specific topic areas.

Third, release letters could be sent in the body of an email instead of the current practice of email attachments and web-based downloads. This would ensure their searchability and ease of reading. Sections that are generic to all letters should be reduced as much as possible and made visually distinct from the portions that require the requestor’s attention.

## Conclusions

The EMA is the only regulator in the world that is routinely releasing part of its holdings, but our experience shows that document release can take considerable time to occur and often only after a lengthy correspondence. Despite the problems, the EMA’s unique efforts should not be undermined. Independent researchers and users should instead support the policy as at present it is the most promising way to address reporting bias in literature. In this report, we have identified areas for improving the system. This case series details our experience with the EMA, but we would like to hear from researchers who approached industry.

## Additional file

Additional file 1:
**Study data extraction sheet.** (xlsx 31 kb)

## Abbreviations

ATD: Access to Documents Service
CHMP: Committee for Medicinal Products for Human Use
CSR: Clinical Study Report
CTD: Common Technical Document
EMA: European Medicines Agency
ICH: International Conference on Harmonization of Technical Requirements for Registration of Pharmaceuticals for Human Use
